# Needle Tip Position and Bevel Direction Have No Effect in the Fluoroscopic Epidural Spreading Pattern in Caudal Epidural Injections: A Randomized Trial

**DOI:** 10.1155/2016/4158291

**Published:** 2016-04-24

**Authors:** Won Kyoung Kwon, Ah Na Kim, Pil Moo Lee, Cheol Hwan Park, Jae Hun Kim

**Affiliations:** ^1^Department of Anesthesiology and Pain Medicine, Konkuk University Medical Center, Konkuk University School of Medicine, Neungdong-ro 120-1, Kwangjin-gu, Seoul 05030, Republic of Korea; ^2^Department of Anesthesiology and Pain Medicine, Wondang OK Pain Clinic, 783 Hoguk-ro, Deogyang-gu, Goyang-si, Gyeonggi-do 10461, Republic of Korea

## Abstract

*Background.* Caudal epidural steroid injections (CESIs) are an effective treatment for pain. If the injection spreads in a specific pattern depending on the needle position or bevel direction, it would be possible to inject the agent into a specific and desired area.* Objectives.* We conducted a prospective randomized trial to determine if the needle position and bevel direction have any effect on the epidural spreading pattern in CESI.* Methods.* Demographic data of the patient were collected. During CESI, the needle position (middle or lateral) and direction (ventral or dorsal) were randomly allocated. Following fluoroscope-guided injection of 4 mL contrast media and 10 mL of injectates, the epidural spreading patterns (ventral or dorsal, bilateral or lateral) were imaged.* Results.* In the 210 CESIs performed, the needle tip position and bevel direction did not influence the epidural spreading patterns at L4-5 and L5-S1 disc levels. A history of Lumbar spine surgery was associated with a significantly limited spread to each disc level. A midline needle tip position was more effective than the lateral position in spreading to the distant disc levels.* Conclusions.* Neither the needle tip position nor the bevel direction affected the epidural drug spreading pattern during CESI.

## 1. Background

A caudal epidural steroid injection (CESI) has been shown to be an effective treatment approach for low back and/or lower extremity pain [1–4]. Some physicians, including the authors of this paper, tend to position the needle tip at the patient's painful side (right or left) to ensure an effective drug spread to the target area of the pain. Using this method, physicians expect that most of the injected drug will spread to the painful side. It is difficult to determine whether this is true due to the lack of studies on the epidural spreading patterns related to needle tip position in CESI.

As spinal discs are located near the ventral epidural space, ventral spread during CESI could be logically presumed to be an effective treatment in patients who have a herniated intervertebral disc (HIVD) or discogenic pain [2, 5, 6]. Gupta et al. [7] and Ackerman III and Ahmad [8] reported that ventral spread of epidurography was associated with improvements in the pain score after interlaminar, transforaminal, and/or caudal epidural injections.

The objective of this study was to determine the majority of the injectate spread in the ventral epidural space if the bevel of the needle is pointed down. If the agent tends to distribute in a certain pattern according to the needle position or bevel direction, it may be possible to inject the agent into a specific area. We tested the hypothesis that the needle position or bevel direction relates to the pattern of epidural spread during CESI.

## 2. Materials and Methods

This prospective randomized study was performed after gaining ethics approval from the Institutional Review Board at our hospital. After obtaining written informed consent, patients scheduled for CESI were included in this study. We collected information regarding age, gender, height, weight, underlying diseases, and any history of lumbar spinal surgery. Exclusion criteria were a lack of informed consent, patients less than 20 years of age, coagulopathy, pregnancy, active infection or pressure sore at the sacral hiatus, or a history of allergic reaction to injected drugs for CESI or any intravascular injection during a procedure.

### 2.1. Description of Interventions

**Figure 1:**
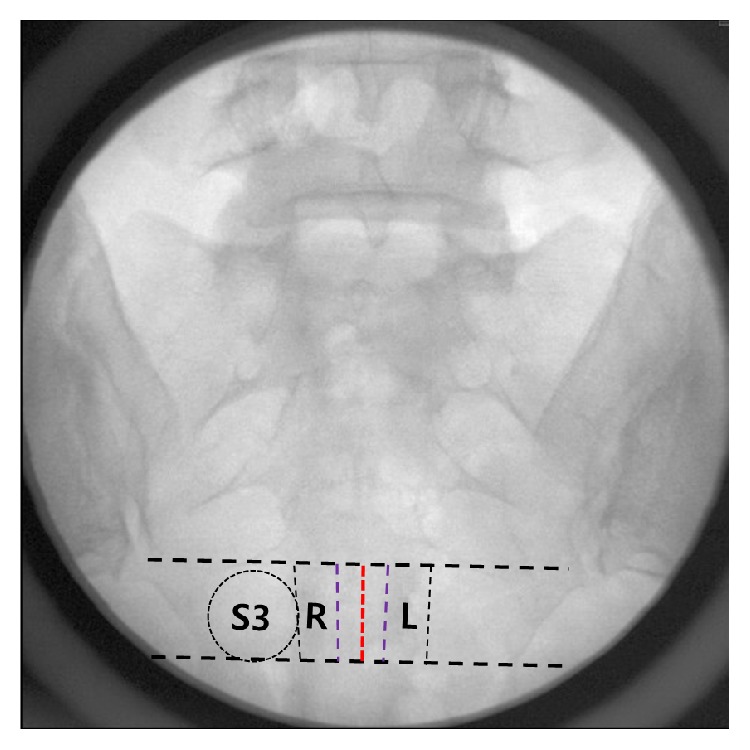
Diagram of the needle tip positions used in this study. S3: ventral foramen of S3, R: needle tip position when injecting in the right side, L: needle tip position when injecting in the left side, red dotted line: needle tip position of midline.

Using a concealed random number table, the needle position (middle or lateral side) and bevel direction (ventral or dorsal) of the needle tip were each randomly allocated. If the lateral side was selected, the needle was inserted into the patient's more painful side (either right or left; Figure 1). All CESIs were performed by the same experienced physician, with over 8000 C-arm fluoroscopy-guided procedures completed. During the CESI, patients were instructed to lie in the prone position, with a 12 cm pillow under the lower abdomen, and were situated so that the median sacral crest of the S2 and S3 levels was midline of the sacrum in fluoroscopic anteroposterior (AP) view. After local infiltration anesthesia at the injection site, an 18 G Tuohy needle (Tae-Chang Industrial Co., Kongju, Korea) was inserted into the epidural space through the sacral hiatus under fluoroscopic guidance. If the middle needle position was selected (group M), the needle was inserted at the midline of the sacral vertebral body (median sacral crest) (Figure 1). If the lateral side needle position was selected (group L), the needle was inserted into the lateral one-third of the sacral canal by fluoroscopic AP view (Figure 1). The needle tip was advanced at the S3 anterior foramen level (Figure 1). Alternatively, if the S3 anterior foramen was not visible, the needle tip was inserted just below the virtual line between both distal ends of the sacroiliac joint.

**Figure 2:**
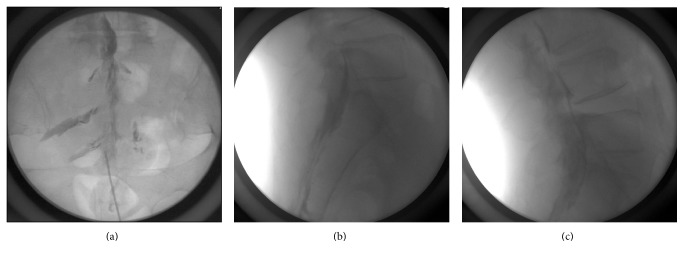
Sample image of the epidural spreading pattern after injection of 4 mL of contrast media using the left side needle tip position ((a) anteroposterior view; (b) lateral view) and (c) after injection of 14 mL of injectates.

Next, the physician turned the bevel of the needle to the selected direction, ventral (group D) or dorsal (group U). After checking for blood aspiration, 4 mL of contrast medium was injected. If intravascular spreading was noted, the patient was excluded from the study, because some of the injected contrast media was not located in the epidural space. After injection of the contrast media, fluoroscopic AP and lateral views of the L2-3, L3-4, L4-5, and L5-S1 disc levels, and the sacral level were obtained and the images were saved (Figures 2(a) and 2(b)). Subsequently, 10 mL of injectates (0.125% levobupivacaine with 2.5–5 mg of dexamethasone and 3 mL of contrast media) was injected, fluoroscopic AP and lateral views were obtained, and these images were saved (Figure 2(c)).

To evaluate the distribution pattern of the injection, the spread of the injected contrast and drugs was confirmed using the fluoroscopic images. The spreading pattern at L4-5 and L5-S1 disc levels was determined in relation to the ventral or dorsal side and left or right side. In addition, the maximum distribution was determined. The spreading pattern in each patient was analyzed by the same experienced pain physician and radiologist, neither of whom was involved in the procedure. If the two physicians did not agree on the results of the analysis, an additional experienced pain physician assessed the fluoroscopic images, and a consensus was reached.

### 2.2. Sample Size Justification

This study was powered to detect a difference in the epidural spreading pattern according to the needle position. In the preliminary investigation, the rate of bilateral spreading at the L5-S1 level was 90% (18 of 20 CESIs) in the central position group and 75% (15 of 20 CESIs) in the lateral position group. To obtain 80% power for analysis, 97 CESIs were required in each group. The only attrition was among patients who received intravascular injection, and the study was halted when the lateral group reached 100 cases.

### 2.3. Statistical Methods

Mean differences in age, height, and weight were analyzed by Student's *t*-test. Proportional differences in gender, lumbar spinal surgery history, needle position, and bevel direction were analyzed by the chi square test. Possible factors affecting the epidural spread, such as bevel direction, needle position, and history of lumbar spine operation, were analyzed by logistic regression analysis. *P* values of less than 0.05 were considered statistically significant.

## 3. Results

There were 229 CESIs performed in this study; 19 cases (8.3%) were excluded because they received an intravascular injection during the CESI. Therefore, 210 CESIs (174 patients) were included in this study. Following the injection of 14 mL of injectates, bilateral epidural spreading occurred in 83.8% of patients (124 of 148) at the L4-5 disc level and 85.4% of patients (170 of 199) at the L5-S1 disc level (Table 1). Ventral epidural spreading occurred in 63.5% of patients (94 of 148) at the L4-5 disc level, increasing to 74.9% of patients (149 of 199) at the L5-S1 disc level.

Needle position comparison was performed between groups M (110 patients) and L (100 patients). There were no significant differences between the two groups regarding age, gender, height, weight, and lumbar spine surgery history (Table 2). There were also no differences between the two groups regarding the pattern of bilateral epidural spreading (right and left side) or ventral epidural spreading (Table 3). In group M, bilateral epidural spreading after injection of 14 mL of injectates was observed in 84.8% of patients (67 of 79) at the L4-5 disc level and in 87.5% of patients (91 of 104) at the L5-S1 disc level (Table 3). Ventral epidural spreading after injection of 14 mL of injectates was observed in 68.4% of patients (54 of 79) at the L4-5 disc level and in 76.9% of patients (80 of 104) at the L5-S1 disc level. There were no statistical differences observed between the epidural spreading to the right, left, or both sides for each needle position (right, middle, and left).

Needle bevel direction comparison was performed between groups U (99 patients) and D (111 patients). There were no significant differences observed regarding age, gender, height, weight, and lumbar spine surgery history between the two groups (Table 4). There were also no differences between the two groups regarding the pattern of bilateral epidural spreading (right and left side) or ventral epidural spreading (Table 5). After caudal epidural injection of 14 mL of injectates, spreading occurred in 59 patients (28.1%) to the L2-3 disc level, 99 patients (47.1%) to the L3-4 disc level, 148 patients (70.5%) to the L4-5 disc level, and 199 patients (94.8%) to the L5-S1 disc level. There were no statistical differences in epidural spreading on the ventral, dorsal, or bilateral sides for each bevel direction (ventral or dorsal).

**Figure 3:**
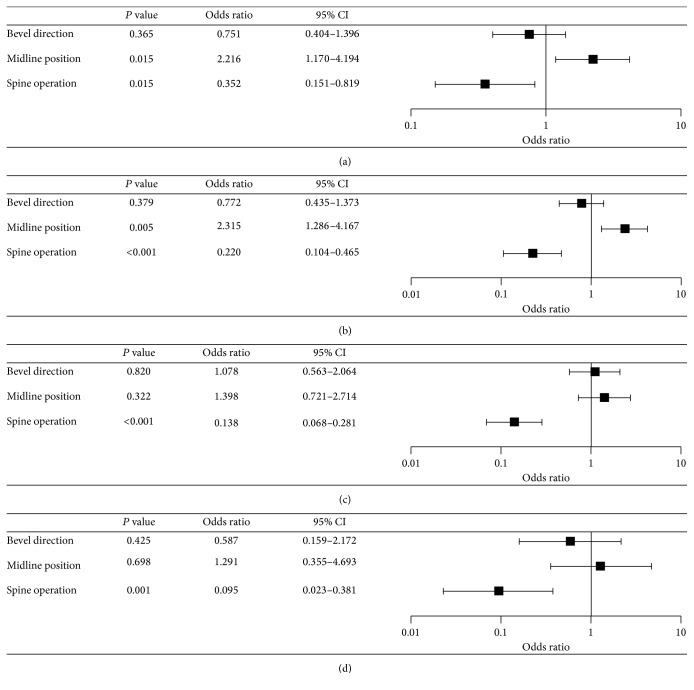
Logistic regression analysis of the epidural spread at (a) L2-3, (b) L3-4, (c) L4-5, and (d) L5-S1 disc levels after injection of 14 mL of injectates.

Using logistic regression analysis, the needle bevel direction (group U or group D) did not correlate to the drug spreading to the L2-3, L3-4, L4-5, and L5-S1 disc levels (Figure 3). The degree of drug spreading to the L2-3 and L3-4 disc levels was significantly higher in group M compared to group L (*P* < 0.05) (Figures 3(a) and 3(b)). The degree of drug spreading at the L2-3, L3-4, L4-5, and L5-S1 disc levels in patients without a history of lumbar spine surgery was significantly higher than that in patients with a history of lumbar spine surgery (*P* < 0.05) (Figure 3).

## 4. Discussion

In the present study, the needle tip position (midline or lateral) during CESI was not related to the epidural spreading pattern at the L4-5 and L5-S1 disc levels. The needle tip position to either the right or left side also did not relate to the epidural spread at the L4-5 and L5-S1 disc levels. Injections in all needle tip positions (right, middle, and left) were characterized by the majority of the drug spreading bilaterally at both the L4-5 and L5-S1 disc levels. Therefore, despite the needle tip being positioned on the lateral side in a patient who has pain in only one side, the epidural spread will most likely be bilateral, rather than unilateral only on the painful side, at the L4-5 and L5-S1 disc levels. These results were inconsistent with our hypothesis that if the needle tip is positioned at a specific side during CESI, the injectate will spread preferentially on that specific side. Lee et al. conducted a study of 22 cases of caudal epidurography following injection of 5 mL of contrast media conducted with the needle positioned toward the affected side in CESI [9]. In contrast to the present study, Lee et al. determined that the average ratio of the contrast media area on the affected side to the total area of epidurography was 73.5%, as measured by pixel count within the area of epidurography [9]. Additionally, they reported that 68.2% of the patients showed a predominant spread to the needle side, 31.8% showed nonpredominant spreading, and 13.6% showed greater spreading to the opposite side [9]. However, in the predominant spread at each nerve root, there were no statistically significant differences between the needle side and opposite side at the L4, L5, and S1 (S2 and S3 were exceptions) nerve roots. Therefore, the predominant spreading may be limited at the sacrum, such as at the S2 and S3 levels described in the results. The number of cases in our study is larger than that of Lee et al.'s study. In our study, we did not investigate the spreading pattern in the sacrum, but instead at the L4-5 and L5-S1 disc levels, which are the most common sites of HIVD [10] and spinal stenosis [11]; therefore, CESI is often conducted at these levels.

The bevel direction (ventral or dorsal) was also not related to the epidural spreading pattern at the L4-5 and L5-S1 disc levels. Therefore, positioning the needle upwards or downwards does not indicate that the epidural spreading will occur on only the dorsal or ventral side. In interlaminar epidural injections, the Tuohy needle bevel direction to the cephalad or caudal side does not relate to spread [12–14] or has only minor effects on the spread [15, 16]. The diameter of the 18-gauge Tuohy needle in this study was 1.2 mm, according to the company brochure. Therefore, the difference of the location of bevel between the up and down side of the needle would be about 1.2 mm. It is believed that this small distance (1.2 mm) may not influence the rate of spreading to the ventral or dorsal side. Despite the different bevel direction of the needle, there were numerous instances in which the drugs spread to the ventral epidural space at the L4-5 level (group U: 59.4%, group D: 67.1%) and the L5-S1 level (group U: 72.6%, group D: 76.9%) after injection of 14 mL of drugs. To inject the drug into the targeted level and side in the epidural space, it was more effective to rotate the Tuohy needle tip 45° and place the catheter to advance the drug into the desired level in interlaminar epidural injections [17, 18]. In CESI, needle tip rotation and catheter insertion at the desired level may also be effective for the injectate to approach the target level. Catheter insertion via sacral hiatus, and drug injection at the target site, is usually performed in epidural neuroplasty [19–21].

In this study, a midline needle tip position was related to spreading to distant disc levels. Drug spread at the L2-3 and L3-4 disc levels was different according to the needle position. If the target level of CESI was at the L2-3 or L3-4 disc level, a midline needle tip position would be helpful in spreading the drug to the target level. After injection of 14 mL of injectates, at L4-5 and L5-S1 disc level, a midline or lateral area needle tip position did not affect the spreading of the injectate. It is believed that the 14 mL injection volume may easily approach the L4-5 and L5-S1 level in most cases, even with a different needle tip position (midline or lateral). Despite the different needle tip positions, bilateral spreading was the most common pattern in CESI. Therefore, we believe that the relatively short distance from the midline needle tip position to a higher spine level of bilateral spreading may have advantages in the degree of drug spread at high disc levels such as L2-3 and L3-4. Lee et al. reported that needle position was related to epidural contrast spreading at the S2 and S3 nerve roots [9]. Therefore, lateral needle tip position may cause leakage through the sacral foramen, and less volume of injectate may spread to the lumbar level rather than with the needle tip in the midline position. We believe that the relatively small volume of injectate in the lateral needle tip position can also reduce the epidural spread to higher levels of the lumbar spine.

In this study, a history of L-spine surgery was a restrictive factor of drug spread at the L2-3, L3-4, L4-5, and L5-S1 disc levels. Adhesions in the epidural space could be triggered by the postoperative wound healing process and scar tissue formation after spinal surgery and have the potential to limit drug distribution in the epidural space [19, 21, 22]. Therefore, epidural adhesion or scarring in patients with a history of L-spine surgery can decrease the cephalic spreading of the drug.

In patients without a history of L-spine surgery, the rate of spreading of 14 mL of injectate was 31.9% at the L2-3, 54.5% at the L3-4, 80.6% at the L4-5, and 98.1% at the L5-S1 disc levels. After 14 mL of epidural injection, in the patients who had no history of L-spine surgery, the injected drugs reached the L5-S1 disc level in almost all cases and the L4-5 disc level in 80.6% of cases. Despite the different needle tip position (midline or lateral), in most cases, 14 mL of CESI epidural drugs can reach the L4-5 and L5-S1 disc levels. Therefore, 14 mL may be a reasonable volume in CESI if the target is the L4-5 or L5-S1 disc level. Kim et al. reported that the epidural spreading level was not significantly changed with subsequent injection from 10 mL to 50 mL in 32 patients of CESI [23]. They said that there was no direct relationship between the volume of injected solution and the number of blocked segments due to the anterior leakage of solution through the sacral foramen. Nevertheless, in previous systematic review, the amount of injected fluid in epidural block was correlated with pain relief among the patients with radicular pain and/or low back pain [24]. Investigation of 20 cases of magnetic resonance epidurography after CESI showed the epidural spread level after injection of 10 mL or 20 mL of injectate [25]. After injection of 10 mL, the injectate was observed at the L3-4 level (56%), the L4-5 level (66%), and the L5-S1 level (89%) [25]. After injection of 20 mL, the injectate was observed at the L3-4 level in all cases [25]. Although the case number was small (9 cases of 10 mL injection and 11 cases of 20 mL injection), the study showed that the volume could affect the highest epidural spreading level in CESI. Although there was no linear correlation between injected volume and spreading level of spine, further investigation about the most effective volume of injectate in CESI will be required.

Cleary et al. reported that a 30° Trendelenburg tilt from the lumbar spine was more effective for cephalic drug delivery in CESI in order to eradicate lumbar lordosis [26]. In our study, a pillow was positioned under the patient's lower abdomen to reduce the lumbar lordosis. The results of the previous study suggest that the dural surface area is one of the primary determinants of epidural longitudinal distribution of local anesthetics [27]. According to this mechanism, conditions that increase abdominal pressure, such as obesity or pregnancy, compress the dural sac, reduce the dual surface area, and reduce the epidural cavity volume so that the final level of the drug injected is higher when the drug is injected into the epidural space [27, 28]. Trendelenburg tilt at the lumbar spine, or a pillow under the abdomen, has been related to a decrease in lumbar lordosis and may increase the abdominal pressure, thereby possibly increasing the spread of the injectate at a high level of the spine.

There are some limitations in this study. Although we calculated the sample size, there was no correlation between needle tip position/direction of bevel and epidural spreading pattern. Nevertheless, we believe that with no correlation in 210 cases, our hypothesized relation between needle tip position or bevel direction and epidural spreading pattern that was tested in this study is likely not useful in clinical cases.

In conclusion, neither the needle tip position (midline or lateral side) nor the Tuohy needle bevel direction impact the epidural drug spreading pattern at the L4-5 and L5-S1 disc levels during CESI. Fourteen milliliters is a reasonable total volume of injectate for the L4-5 or L5-S1 disc pathology in CESI. Patients with a history of L-spine surgery may experience limited epidural spreading to most lumbar disc levels. However, a midline needle tip position may be helpful in epidural drug spreading to the distant disc levels in CESI.

## Figures and Tables

**Table 1:** The rates of bilateral and ventral drug spread at L4-5 and L5-S1 disc levels after injection of 4 mL of contrast medium or 14 mL of injectates.

Injection drug	Bilateral spread		Ventral spread	
	L5-S1 level	L4-5 level	L5-S1 level	L4-5 level
4 mL of contrast	115/175 (65.7%)	35/56 (62.5%)	101/175 (57.7%)	24/56 (42.9%)
14 mL of injectates	170/199 (85.4%)	124/148 (83.8%)	149/199 (74.9%)	94/148 (63.5%)

The data is expressed as “the number of cases with spreading/total number of cases” (percent of cases with spreading).

**Table 2:** Demographic data of the patients treated in group M and group L.

	Group M (*n* = 110)	Group L (*n* = 100)	*P* value
Age	63.4 ± 14.9	59.8 ± 16.0	0.092
Gender (female/male)	73/37	55/45	0.092
Height	159.8 ± 8.4	161.0 ± 10.5	0.383
Weight	61.0 ± 10.6	62.1 ± 11.8	0.438
Lumbar spine operation history	32	18	0.059
DM	17	8	0.096
HTN	49	37	0.267

Individuals from group M had their needle inserted at the midline of the sacral vertebral body.

Individuals from group L had their needle inserted into the lateral one-third of the sacral canal.

Data is expressed as “mean ± SD” or “number of individuals.”

DM: diabetes mellitus; HTN: hypertension.

**Table 3:** The rates of bilateral and ventral epidural spread in groups M and L after injection of 4 mL of contrast medium or 14 mL of injectates.

Injection drug	Epidural spreading	Group M (*n* = 110)	Group L (*n* = 100)	*P* value
4 mL of contrast medium	Bilateral spreading at L5/S1	66/93 (71.0%)	49/82 (59.8%)	0.119
	Bilateral spreading at L4/5	21/32 (65.6%)	14/24 (58.3%)	0.577
	Ventral spreading at L5/S1	58/93 (62.4%)	43/82 (52.4%)	0.185
	Ventral spreading at L4/5	17/32 (53.1%)	7/24 (29.2%)	0.073

14 mL of injectates	Bilateral spreading at L5/S1	91/104 (87.5%)	79/95 (83.2%)	0.386
	Bilateral spreading at L4/5	67/79 (84.8%)	57/69 (82.6%)	0.717
	Ventral spreading at L5/S1	80/104 (76.9%)	69/95 (72.6%)	0.486
	Ventral spreading at L4/5	54/79 (68.4%)	40/69 (58.0%)	0.191

Individuals from group M had their needle inserted at the midline of the sacral vertebral body.

Individuals from group L had their needle inserted into the lateral one-third of the sacral canal.

The data is expressed as “the number of cases with spreading/total number of cases” (percent of cases with spreading).

**Table 4:** Demographic data of the patients treated in group U and group D.

	Group U (*n* = 99)	Group D (*n* = 111)	*P* value
Age	60.5 ± 16.7	62.7 ± 14.4	0.313
Gender (female/male)	61/38	67/44	0.852
Height	160.8 ± 10.2	160.1 ± 8.8	0.576
Weight	62.6 ± 9.9	60.5 ± 12.2	0.199
Lumbar spine operation history	24	26	0.889
DM	13	12	0.604
HTN	38	48	0.475

Individuals from group U had the bevel of the needle headed to the dorsal side.

Individuals from group D had the bevel of the needle headed to the ventral side.

Data is expressed as “mean ± SD” or “number of individuals.”

DM: diabetes mellitus; HTN: hypertension.

**Table 5:** The rates of bilateral and ventral epidural spread in groups U and D after injection of 4 mL of contrast medium or 14 mL of injectates.

Injection drug	Epidural spreading	Group U (*n* = 99)	Group D (*n* = 111)	*P* value
4 mL of contrast medium	Bilateral spreading at L5/S1	55/83 (66.3%)	60/92 (65.2%)	0.232
	Bilateral spreading at L4/5	16/25 (64.0%)	19/31 (61.3%)	0.835
	Ventral spreading at L5/S1	44/83 (53.8%)	57/92 (62.0%)	0.232
	Ventral spreading at L4/5	10/25 (40.0%)	14/31 (45.2%)	0.698

14 mL of injectates	Bilateral spreading at L5/S1	78/95 (82.1%)	92/104 (88.5%)	0.204
	Bilateral spreading at L4/5	58/69 (84.1%)	66/79 (83.5%)	0.933
	Ventral spreading at L5/S1	69/95 (72.6%)	80/104 (76.9%)	0.486
	Ventral spreading at L4/5	41/69 (59.4%)	53/79 (67.1%)	0.334

Individuals from group U had the bevel of the needle headed to the dorsal side.

Individuals from group D had the bevel of the needle headed to the ventral side.

The data is expressed as “the number of cases with spreading/total number of cases” (percent of cases with spreading).

## Additional Points

The authors conducted a prospective randomized trial to determine if the needle position and bevel direction have any effect on the epidural spreading pattern in caudal epidural steroid injections (CESIs). In the 210 CESIs performed, neither the needle tip position nor the bevel direction affected the epidural drug spreading pattern during CESI. Patients with a history of lumbar spine surgery may experience limited epidural spreading to the different lumbar disc levels. A midline needle tip position may promote epidural drug spreading to the distant disc levels in CESI.
